# External jugular venous aneurysm—a case report

**DOI:** 10.1093/jscr/rjae494

**Published:** 2024-08-08

**Authors:** Nihal Sogandji, Guglielmo La Torre, Diane Hildebrand

**Affiliations:** School of Clinical Medicine, University of Cambridge, Addenbrooke's Hospital, Hills Rd, Cambridge CB2 0SP, United Kingdom; Department of Vascular Surgery, Addenbrooke’s Hospital, Cambridge University Hospitals National Health Service Foundation Trust, Hills Rd, Cambridge CB2 0QQ, United Kingdom; Department of Vascular Surgery, Addenbrooke’s Hospital, Cambridge University Hospitals National Health Service Foundation Trust, Hills Rd, Cambridge CB2 0QQ, United Kingdom

**Keywords:** venous aneurysm, external jugular vein

## Abstract

Aneurysms of the external jugular vein (EJV) are uncommon vascular malformations. Due to their rarity, it is currently unclear what the risk of complications are and whether surgical management should be offered. The risks associated with the surgery need to be balanced with the risk of complications from the malformation. We present the case of a young woman who presented with a painful erythematous neck swelling who was found to have an aneurysm of the EJV with thrombophlebitis. This was successfully treated with surgical excision. We discuss current evidence for treatment of neck vein aneurysms and pitfalls.

## Introduction

Venous aneurysms are extremely uncommon vascular malformations described as focal dilatations which have a single-channel communication with a normal, non-varicose vein [1]. There is no consensus on how to manage these lesions, with asymptomatic cases usually managed conservatively following consideration of the risks of rupture, bleeding and venous thromboembolism (VTE) from the aneurysm as well as cosmetic concerns. Surgical excision is possible, but the risks would include bleeding, thrombophlebitis, damage to the nerves, wound complications, and recurrence [2]. We present the case of a 37-year-old woman who had an aneurysm of the external jugular vein (EJV) that was treated surgically.

## Case report

**Figure 1:**
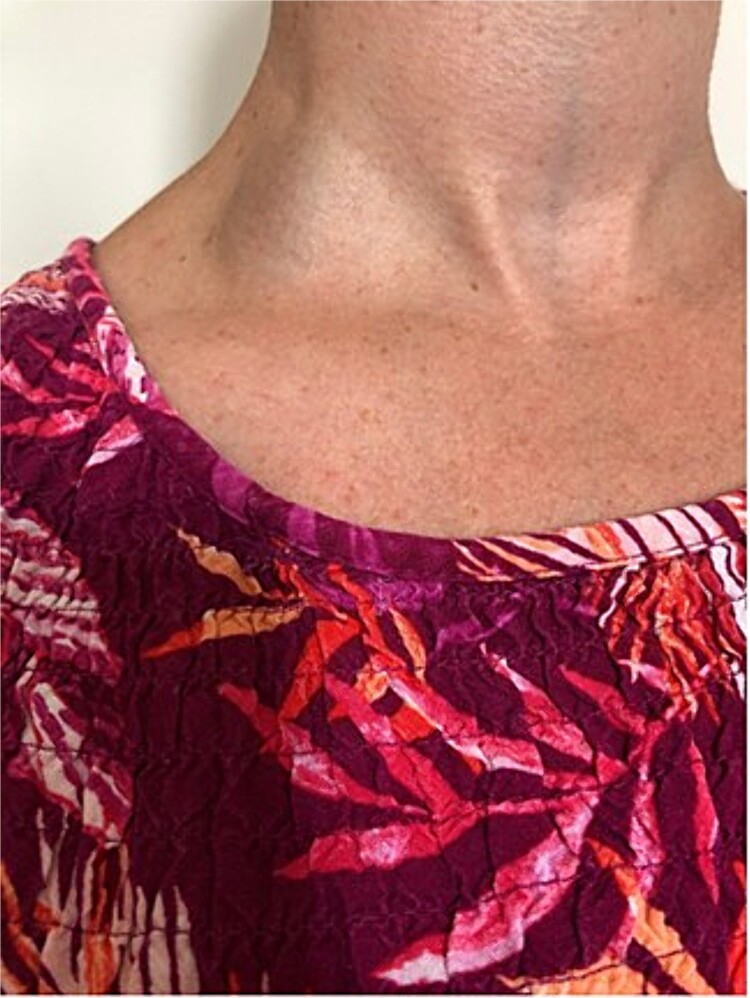
EJV aneurysm without Valsalva manoeuvre.

**Figure 2:**
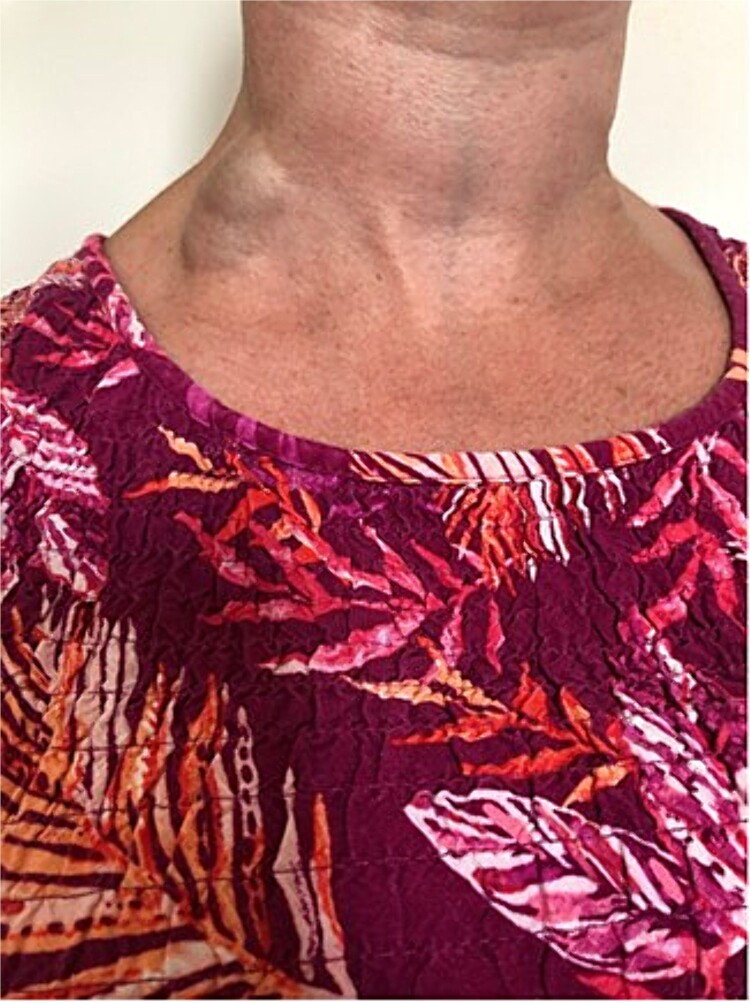
EJV aneurysm with Valsalva manoeuvre.

A 37-year-old female patient was referred to the vascular surgical department with a chronic swelling in the posterior triangle of the neck, which had become painful and erythematous. This had been present for a number of years, first noticed after a bout of cough approximately a decade prior. As it had not troubled the patient, this had never been investigated. Recently, however, after an episode of heavy lifting in the garden, the patient noted new erythema and pain over the swelling. A duplex scan of the neck was carried out and showed extensive non-occlusive acute thrombus in the EJV. The internal jugular vein and subclavian vein were patent. Clinical examination with and without Valsalva manoeuvre (as per Figs. 1 and 2) confirmed the aneurysmal nature of the vessel. Due to the rare nature of the vascular anomaly, a multidisciplinary opinion was sought. Management with anticoagulation or surgical excision were considered. This venous aneurysm was generally deemed low risk in terms of consequently leading to a pulmonary embolism, however, the option of resection under local anaesthetic was offered to the patient as this was deemed to have a better risk profile in the long term. Under local anaesthetic, a transverse incision was performed over the aneurysm. This was dissected and all feeding vessels ligated with 2-0 Vicryl. The patient was discharged home with a course of anticoagulation with Rivaroxaban for 6 weeks, as she was due to undergo a long-haul flight in the coming days post-operatively. Post-operatively, she made a good recovery, although she does experience paraesthesia of the skin in the surrounding region.

## Discussion

Approximately 500 cases of venous aneurysms have been reported [2] to date, and, to our knowledge, no standardized guidelines for management exist.

The exact pathophysiology remains unknown; however, weakened vessel walls due to degeneration or congenital defects, inflammation, or high venous pressure have been proposed as possible mechanisms [3]. Weakening of the elastic lamina and aberration of matrix metalloproteinases expression has also been proposed [2]. Venous aneurysms are classified as primary or secondary based on whether there is history of relevant trauma, or underlying pathology such as connective tissue disorders or arteriovenous malformations [2]. They are most commonly reported in the popliteal veins [2] but have also been seen in the superficial and deep leg veins, veins of the thorax, abdomen, and head and neck veins. The latter being especially likely due to the superficial, exposed, location of the jugular veins, which also predisposes to aneurysmal rupture from trauma. These aneurysms can also be caused iatrogenically from procedures such as placement of venous catheters [4].

Symptomatic patients tend to be treated with surgery; however, due to the rarity of these vascular lesions, there are no established standards of management. Risks of conservative management such as VTE and rupture are balanced against surgical risks, which vary depending on the anatomical location and patient factors [2]. The risk of embolism is low with aneurysms of the jugular veins and asymptomatic cases usually managed conservatively. However, reasons for considering surgical management include concerns relating to appearance, pain, or thrombophlebitis within the aneurysm [5]. The exact intervention depends on the type of aneurysm, with exclusion bypass preferred for fusiform aneurysms, whereas surgical resection and ligation tends to be done for saccular aneurysms [6], even though no standard guidelines for treatment exist.

Following a review of the literature, Teter et al. [7] propose any symptomatism as a clear indication for surgical management. In cases where surgical management has been preferred, post-operative anticoagulation regimens are inconsistent, varying from 3 to 6 months in duration [2, 8]. Furthermore, the medical literature seems to show that anticoagulation alone may not be enough to stop the occurrence of VTE, with rates of VTE with medical management alone reported as high as 60% [2]. Endovascular treatment may be an attractive option to mitigate the risks of surgical excision. Pandey *et al*. [9] report a case of successful endovascular embolization although anatomical position would require careful consideration, especially for large aneurysms.

It is currently unclear whether surveillance should play a role in the management of asymptomatic aneurysms.

There are ~10 cases of EJV aneurysms reported in the medical literature [10]. In many of these cases, the patient presented with a few months’ history of a neck mass, and surgical management was preferred due to cosmetic concerns noted by the patient in spite of the benign nature of the lesion and the low risk of complications like VTE [3–6, 9, 10].

Being an extremely rare cause of cervical swelling, other differential diagnoses need to be considered and ruled out. These include cervical lymphadenopathy, malignancy of nearby tissue, and cysts. An EJV aneurysm is typically seen as a compressible swelling superficial to the sternocleidomastoid muscle, which becomes more prominent on breath holding [6] and changes size with posture and the Valsalva manoeuvre [9]. A definitive diagnosis requires Doppler ultrasound imaging.

## Conclusion

To conclude, aneurysmal malformation of the EJV is rare. Due to its rarity, it is unclear how to best manage this condition; however, our case shows that surgical repair can be feasible and safe in symptomatic disease.
